# Electric Field-Driven Melt Jetting Polycaprolactone Rotational Printing of Fully Degradable Vascular Stents and Mechanical Characterization

**DOI:** 10.3390/polym18010074

**Published:** 2025-12-26

**Authors:** Yanpu Chao, Fulai Cao, Hao Yi, Shuai Lu, Chengyan Zhang, Hui Cen, Zhongfu Liu, Yihang Yao, Xiaobo Zhao

**Affiliations:** 1 School of Mechatronics Engineering, Xuchang University, Xuchang 461000, China; 2 State Key Laboratory of Mechanical Transmission, Chongqing University, Chongqing 400044, China

**Keywords:** vascular scaffolds, electric field-driven, rotational deposition, mechanical behavior, fracture mechanisms

## Abstract

Addressing technical challenges in personalized fabrication and mechanical regulation of bioresorbable vascular scaffolds, this study pioneers an electric field-driven melt jetting rotational printing technique to fabricate polycaprolactone (PCL) scaffolds (Ø3–8 mm). Multiscale characterization confirms a rhombic mesh macrostructure with uniform fibers and fusion-enhanced nodal junctions, demonstrating synergistic control of electrohydrodynamic forces and surface tension over microfiber deposition. Mechanical testing reveals triphasic tensile behavior (elastic-plastic-fracture), where 5 mm scaffolds exhibit 38% enhanced peak load due to superior interfacial bonding and densified geometry, while 8 mm counterparts suffer premature failure from structural weakening. Fractography identifies brittle fracture initiation at stress-concentrated nodes versus ductile dominance in straight segments, confirming co-regulation by intrinsic material properties and architecture. Compression tests demonstrate characteristic load-holding-recovery behavior, with 20% increased load-bearing capacity and enhanced elastic recovery in larger scaffolds. This work establishes a structure–property correlation framework for optimizing degradable vascular implants, providing novel methodologies and theoretical foundations for clinical compatibility.

## 1. Introduction

Coronary artery disease (CAD), among the most prevalent cardiovascular pathologies, primarily stems from atherosclerosis—the accumulation of cholesterol-rich plaques within arterial walls [1]. This process induces arterial narrowing and stiffening, which restricts or even blocks blood flow [2,3], ultimately causing myocardial damage. Current therapeutic interventions include pharmacotherapy, surgery, and catheter-based stent implantation. Percutaneous coronary intervention (PCI) with stenting is recognized as the most effective technique for CAD management, widely adopted due to its minimally invasive nature and enhanced procedural safety [4,5]. During PCI, a vascular stent is deployed at the lesion site to dilate stenotic/occluded vessels and scaffold the arterial wall, thereby restoring physiological blood perfusion [6].

Biodegradable polymeric vascular scaffolds (BPVS), representing the fourth-generation endovascular implants [7], offer distinct advantages over conventional bare-metal stents (BMS) [8] and drug-eluting stents (DES) [9]. These include low mass, transient radial support, exceptional biocompatibility, superior processability, and controllable biodegradation [10]. Critically, BPVS eliminate complications associated with permanent metallic implants while minimizing physiological impact, establishing them as a promising alternative for safer interventional strategies.

An ideal biodegradable polymeric vascular scaffold (BPVS) must possess essential biomechanical properties including sufficient radial strength, longitudinal flexibility, high radial expandability, fatigue resistance, and optimal wall apposition capability [11]. These characteristics enable effective scaffolding of stenotic segments, mitigation of vascular elastic recoil, and fulfillment of clinical requirements. However, current clinically deployed BPVS exhibit critical limitations in mechanical integrity and device compliance. Consequently, enhancing the radial strength and conformability of polymeric scaffolds has emerged as a pivotal research focus within the field.

Current commercial vascular scaffolds predominantly employ networked tubular architectures. Optimization of strut and connector geometries significantly enhances mechanical performance [12]. Experimental and computational analyses demonstrate that geometric parameters-including strut/connector width, length, thickness, and cross-sectional profiles -critically influence radial strength and compliance characteristics. Furthermore, salt-bath isothermal heat treatment of polymeric scaffolds substantially improves radial stiffness and peak radial load capacity while eliminating residual strains. This enables safer tolerance of bending deformation (curvature > 0.15 mm^−1^) during deployment [13].

Fabrication methodologies for fully bioresorbable vascular scaffolds encompass braiding [14], injection molding [15], laser cutting [16], electrospinning [17], and additive manufacturing (3D printing), with the latter emerging as the dominant technique due to its superior manufacturing efficiency, material economy, capacity for complex 3D architectures, and customizability. Key 3D printing approaches include selective laser melting (SLM) [18], stereolithography (SLA) [19], and fused deposition modeling (FDM) [4]. The FDM process extrudes molten polymers through a heated nozzle to deposit filaments layer-by-layer per digital models, enabling precise scaffold fabrication. Crucially, FDM eliminates material thermal degradation and high surface roughness (Ra > 10 μm) associated with SLM’s elevated processing temperatures, while overcoming SLA’s limitation to photopolymerizable materials, thereby enabling versatile thermoplastic usage (e.g., PCL/PLA/PGA) and avoiding post-curing cytotoxicity risks.

Despite its advantages, conventional FDM faces critical challenges in vascular scaffold fabrication. The complex spatial lattice architecture necessitates extensive support structures (occupying > 40% print volume) to prevent sagging during deposition, complicating post-processing and reducing manufacturing efficiency [20]. Support removal frequently induces micro-damage or deformation in delicate scaffold features, compounded by suboptimal surface finish (Ra > 15 μm) and limited resolution in strut/connector dimensions (>200 μm minimum feature size). These constraints impede precise replication of biomechanically optimized designs [21], necessitating fundamental process re-engineering for vascular applications.

Electric field-driven melt jet deposition 3D printing technology represents an innovative process utilizing self-induced electrostatic fields for high-resolution micro/nanoscale printing [22]. This technique employs a high-voltage electric field applied to a conventional FDM printhead, where synergistic actions of electrostatic forces, surface tension, and viscous stresses [23] elongate molten polymer at the nozzle tip into a Taylor cone. Leveraging the cone’s necking effect, continuous jet deposition of ultrafine fibers (diameter < 10 μm) enables high-efficiency, low-cost fabrication of complex micro/nanoscale features. Peng et al. successfully employed electric field-driven jet deposition 3D printing technology to fabricate a PCL thin-wall tubular grid structure, demonstrating the feasibility of printing polymer vascular stent microstructure with this technology [24].

However, the existing studies lack theoretical modeling for the printing construction of vascular stent geometries, and there is a lack of systematic research and analysis on the influencing laws of the shape of the formed mesh, the wall thickness of the tube, the size of the connecting ribs, and the mechanical properties of the stent. Therefore, it is impossible to achieve precise and effective control.

Based on this, our research team has constructed an effective theoretical model for the printing construction of vascular stent geometries. By optimizing the spiral direction, deflection angle, pitch size, and position offset of the printing deposition, the effective control of the shape of the formed mesh, the wall thickness of the tube, and the size of the connecting ribs of the polymer vascular stent can be achieved. This article is based on the previously established printing construction theoretical model, ultrafine extruded fibers are precisely deposited onto rotating mandrels, enabling precise control over scaffold mesh geometry, diameter (3–8 mm), and wall thickness (100–300 μm). Through systematic parametric investigation, we elucidate the mechanistic influences of electric field voltage, printing temperature, mandrel diameter, rotational speed, and scaffold architecture on mechanical properties. This approach addresses critical limitations in radial strength and compliance of degradable polymeric scaffolds while fulfilling clinical requirements for streamlined manufacturing, cost efficiency, and patient-specific customization.

## 2. Materials and Methods

### 2.1. Experimental Materials

Polycaprolactone (PCL; CAS No.: 24980-41-4) polymer particles supplied by Shandong USOLF (Linyi City, China) were employed as the printing feedstock in this study. PCL has a chemical formula of (C_6_H_10_O_2_)_n_ and an average molecular weight of 50,000 g/mol. As a non-cytotoxic biomaterial approved by the FDA for medical implant applications, it exhibits excellent biocompatibility and biodegradability, with a hemolysis rate < 5% that complies with medical material standards. Its in vivo complete degradation period ranges from 2 to 4 years. Physiochemically, PCL has a melting point of 65 °C, a density of 1.146 g/mL at 25 °C, and a melt viscosity of 11.25 dL/g. Slow cooling yields a crystallinity of approximately 45–50%. Mechanically, it possesses a yield stress of 17.5 MPa, Young’s modulus of 470 MPa, tensile strength of 25 MPa, and elongation at break of 300–900%. With superior ductility and a fracture toughness of 1.5–3.0 MPa·m^1/^^2^, PCL demonstrates mechanical properties analogous to those of ligament tissue.

### 2.2. Principle of Technology

**Figure 1:**
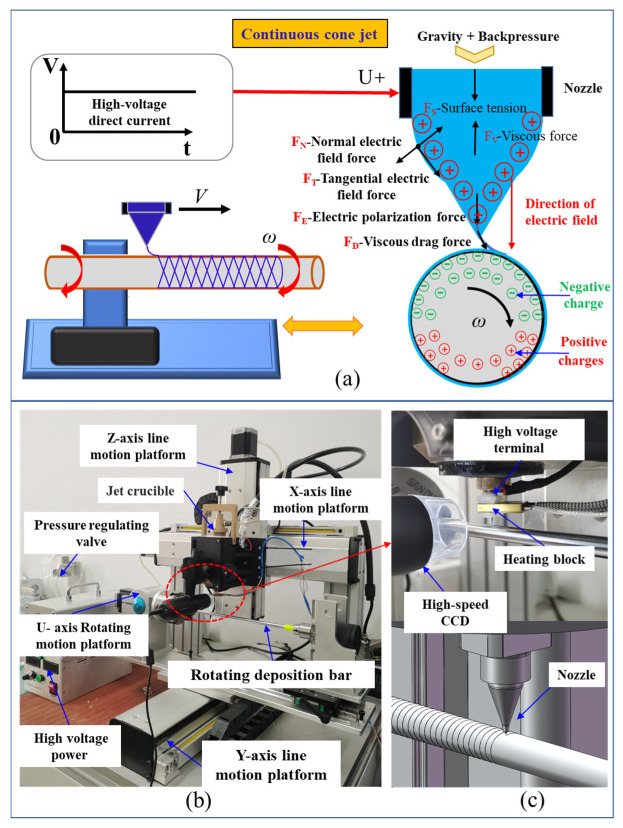
Formation mechanism and experimental setup for electric field-driven melt jetting polycaprolactone rotational printing technique of vascular scaffolds: (**a**) Formation mechanism; (**b**) Experimental setup; (**c**) Enlarged cross-sectional schematic of microfiber deposition on the rotating mandrel surface.

Electric field-driven melt jetting rotational printing technique is a novel process that leverages self-induced electrostatic fields to achieve high-resolution micro/nano-scale jet printing. As illustrated in Figure 1a: A conductive nozzle connected to the positive terminal of a high-voltage DC power supply ejects polymer material. Under the combined influence of gravity and gas backpressure, the material forms a meniscus at the nozzle tip. Upon electrification, the material becomes polarized, accumulating positive charges at the meniscus. When the nozzle approaches the deposition mandrel, electrostatic induction occurs between the conductive nozzle and the mandrel, resulting in negative charge accumulation on the upper semicircle of the mandrel and positive charges on the lower semicircle. The interaction between the positively charged polymer meniscus and the negatively charged upper mandrel region generates an electric field. Under the combined actions of electrostatic forces, surface tension, and other field-induced stresses, the material at the nozzle tip elongates into a Taylor cone. As voltage increases, charge density escalates at the cone apex. When the resultant electrostatic forces comprising the normal electric field force (*F_N_*), tangential electric field force (*F_T_*), and electric polarization force (*F_E_*) exceed the resistive forces of surface tension (*F_S_*) and viscous drag (*F_V_*), the polymer is ejected as a micro-scaled jet. This jet deposits onto a mandrel rotating at angular velocity ω. The viscous drag force (*F_D_*) induced by mandrel rotation further elongates the jet into microfibers. Subsequently, the fibers form helical architectures upon deposition. By synchronizing nozzle translation with mandrel rotation while modulating jetting parameters, precise control over deposition trajectory characteristics including pitch and helix angle is achieved. This capability establishes a foundation for fabricating vascular scaffolds with tailored microarchitectures.

The experimental system designed based on the principles of Electric field-driven melt jetting rotational printing technique is illustrated in Figure 1b. This modular system comprises seven core components: a high-voltage power supply, multi-axis motion control platform, precision nozzle assembly, material crucible, pressure regulation module, high-speed charge-coupled device (CCD) imaging system, and temperature control unit, each serving distinct functional roles in the fabrication process. The high-voltage direct-current (DC) power supply establishes the electrostatic field essential for jet formation. By generating an electric potential gradient between the conductive nozzle and deposition mandrel, it enables the molten polymeric material to overcome intrinsic surface tension and viscous resistance, facilitating the elongation of charged fluid filaments into stable electrospray jets. Adjustable voltage outputs (0–30 kV) allow real-time modulation of jet morphology from dripping to cone-jet regimes—and deposition kinetics, providing precise control over material ejection rates and fiber laydown patterns.

The motion system integrates a three-axis (X-Y-Z) linear stage and a rotational U-axis stage. These orthogonal linear actuators (positioning accuracy: ±10 μm) enable planar (X-Y) and vertical (Z) positioning of the nozzle relative to the mandrel. Precise control via a digital motion controller allows dynamic adjustment of inter-component spacing, thereby regulating deposited layer thickness, fiber alignment angles, and spatial deposition coordinates (X, Y, Z). The mandrel is mounted on a high-speed U-Axis rotational stage (angular velocity range: 0–500 rpm), which imparts both circumferential motion and viscous drag forces to the deposited fibers. This rotational action ensures uniform helical fiber distribution on the mandrel surface and enhances fiber stretching via shear forces, enabling the fabrication of microfibers with diameters down to 100 nm.

The nozzle serves as the critical interface for material extrusion, featuring a tapered orifice (diameter: 100–500 μm) machined from conductive stainless steel. Its geometric precision (surface roughness Ra < 0.8 μm) and electrical conductivity are paramount for maintaining stable Taylor cone formation and jet trajectory control. Any manufacturing tolerances in nozzle geometry can lead to asymmetric charge distribution, thereby compromising deposition accuracy and fiber uniformity. A closed-loop pressure control system, comprising a pneumatic pump and proportional-integral-derivative (PID) controller, maintains stable material flow through the nozzle. By regulating the backpressure (0–50 kPa) applied to the material crucible, this module ensures consistent extrusion velocities (0.1–10 mm/s), preventing issues such as dripping, clogging, or pulsatile flow. Real-time pressure feedback loops are integrated to compensate for temperature-dependent viscosity variations in the molten polymer. A high-speed CCD camera (frame rate: 1000 fps) equipped with a macro lens provides real-time visualization of key process parameters: Imaging of the nozzle-tip meniscus allows quantification of critical parameters, including cone angle and meniscus height. High-resolution videos enable tracking of jet breakup length, bead formation frequency, and deposition impact velocity, providing empirical data for process optimization.

A resistive heating element with proportional temperature control (accuracy: ±1 °C) maintains the polymeric material in a stable molten state within the crucible. The heating profile is tailored to the material’s thermal properties, ensuring a consistent melt viscosity (η = 100–5000 mPa·s) that balances jet stability and fiber stretchability. Overheating is mitigated via a forced-air cooling system, preventing thermal degradation of the polymer during prolonged operation.

During the experimental procedure, the material is first heated to a molten state via a heating module, and then the molten material is fed into the nozzle through a pressure regulating valve. Under the influence of a high-voltage electric field, the molten material forms charged jets that deposit onto the rotating mandrel. Precise construction of vascular scaffolds is achieved by adjusting electric field parameters, the motion trajectory and speed of the motion platform, and nozzle ejection parameters. Figure 1c shows a magnified schematic of microfiber deposition on the rotating mandrel surface. The entire experimental system exhibits high flexibility and controllability, providing strong support for the application of electric-field-driven melt electrospray rotational deposition technology in the field of vascular stent manufacturing.

### 2.3. Morphological Characterization Testing Methods

Microscopic morphology and dimensional characteristics of the samples were characterized via a Keyence VHX-7100 deep-field microscope(KEYENCE CORPORATION, Osaka, Japan). Representative regions across each sample were selected, and surface/cross-sectional micrographs were acquired at magnifications of 100×, 300×, and 500×. Morphological uniformity, pore distribution, and other key features were systematically analyzed. Core dimensional parameters (e.g., diameter, thickness, and pore size) were measured repeatedly at ≥5 fields of view per sample, with mean values and standard deviations (±s) computed to guarantee the accuracy and reliability of the test results.

### 2.4. Mechanical Property Testing Methods

The mechanical property test uses an EDIBURG force tester, which is equipped with a high-precision force sensor and a displacement measuring device, capable of collecting mechanical data and displacement changes in real-time. The diameters of the vascular stent samples tested are 3 mm, 4 mm, 5 mm, and 8 mm, respectively. The tensile test is conducted at a tensile rate of 3 mm/s until fracture; the planar compression test is carried out at a compression rate of 0.7 mm/s, with real-time recording of the compression force and compression deformation. Three samples are tested for each diameter of the vascular stent to ensure the reliability of the test.

In the testing and calculation of the radial support strength and tensile strength of PCL vascular stents, the specific calculation methods (Formulas: (1) and (2)) are as follows in combination with international standards (ISO 25539-2, ASTM F2606) [25,26] and the experimental scenario of this study:

Tensile Strength-$\sigma_{t}$ (MPa):

(1) $$\sigma_{t}=\frac{{\mathrm{Ft}}_{\max}}{A_{t}}=\frac{{4*\mathrm{Ft}}_{\max}}{\pi *({D_{o}}^{2}-{D_{i}}^{2})}$$

where *Ft_max_* is the maximum breaking force recorded in the tensile test (unit: *N*); *A_t_* is the effective tensile cross-sectional area of the stent (unit: mm^2^); the effective tensile cross-sectional area *A_t_ = π × (D_o_*^2^ *− D_i_*^2^*)/*4 (*D_o_* is the outer diameter of the stent, *D_i_* is the inner diameter of the stent, both accurately measured by a Keyence microscope).

Radial Support Strength *R_s_* (kPa):

(2) $$R_{s}=\frac{{\mathrm{Fp}}_{\max}}{A}*10^{-3}=\frac{{\mathrm{Fp}}_{\max}}{\pi *D*L}*10^{-3}$$

where *Fp_max_* is s the maximum bearing force recorded during the radial compression of the stent (unit: *N*), which is collected in real time by an EDIBURG force tester; *A* is the effective radial support area of the stent (unit: mm^2^); for hollow tubular stents, *A = π × D × L* (*D* is the nominal diameter of the stent, *L* is the effective working length of the stent, both measured by a Keyence microscope); Multiplying by 10^−3^: converts the unit from N/mm^2^ (MPa) to kPa (1 MPa = 1000 kPa).

## 3. Experimental Results and Measurement Analysis

Experimental parameters were configured as follows in Table 1: Stainless steel mandrels with diameters of 8 mm, 5 mm, 4 mm, and 3 mm served as deposition substrates. A Musashi nozzle (inner diameter: 300 μm) was maintained at 90 °C (±0.5 °C), while the material reservoir crucible was heated to 130 °C (±0.8 °C). A constant backpressure of 15 kPa (±0.3 kPa) and applied voltage of 1.80 kV (±0.05 kV) enabled stable jetting. The nozzle-to-mandrel vertical distance was fixed at 2.00 mm (±20 μm) with controlled axial translation at 37.6 mm/s (±0.5 mm/s) and mandrel rotation at ω = 16 rad/s (±0.2 rad/s).

**Figure 2:**
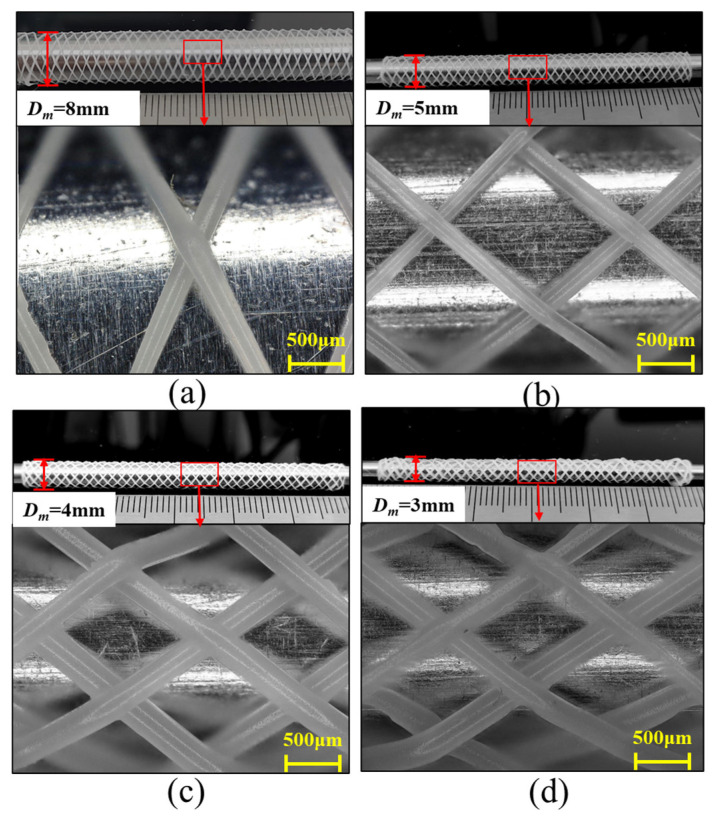
Results of vascular scaffolds printed using deposition mandrels with different diameters: (**a**) 8 mm, (**b**) 5 mm, (**c**) 4 mm, (**d**) 3 mm.

In the experiments, four vascular scaffolds with distinct diameters were fabricated via electric field-driven melt jetting polycaprolactone rotational printing, with the resultant structures presented in Figure 2. Morphologies of the printed scaffolds corresponding to mandrel diameters of 8 mm, 5 mm, 4 mm, and 3 mm were captured using a Keyence VHX-7100 deep-field microscope (KEYENCE CORPORATION, Osaka, Japan) at 300× magnification, as shown in Figure 2a, 2b, 2c and 2d, respectively. Macroscopically, all scaffolds exhibited regular grid-like architectures, confirming the system’s precision in controlling the deposition paths of polymer microfibers to achieve predefined geometries. Microscopically, robust bonding at fiber junctions, consistent fiber diameters, and absence of defects (e.g., fractures or excessive fusion) demonstrated optimized synergy between electrostatic forces and surface tension during jetting, ensuring stable and continuous material deposition. For the scaffold fabricated on an 8 mm mandrel (Figure 2a), relatively sparse fiber packing and large inter-fiber pores were observed. The scaffold produced with a 5 mm mandrel (Figure 2b) displayed moderate fiber density intermediate between those in Figure 2a,c, suggesting balanced mechanical integrity and enhanced tissue compatibility. The scaffold produced with a 5 mm mandrel (Figure 2b) displayed moderate fiber density intermediate between those in Figure 2a,c, suggesting balanced mechanical integrity and enhanced tissue compatibility. The densest architecture with maximal fiber interlacement density was attained using the 3 mm mandrel (Figure 2d), a configuration potentially optimal for cellular growth and vascular tissue regeneration, though requiring further mechanical validation for targeted vascular applications.

Comparative analysis of the structures of four vascular scaffolds with different diameters fabricated via deposition printing reveals that within a certain range, as the scaffold diameter decreases, the fiber filament arrangement remains neat and ordered, while the density of fiber weaving gradually increases. This trend suggests that smaller-diameter scaffolds may be more conducive to cell attachment, proliferation, and tissue ingrowth. However, in practical applications, their mechanical properties and the specific requirements of the target blood vessels must still be considered.

### 3.1. Outer Contour Dimensional Analysis

**Figure 3:**
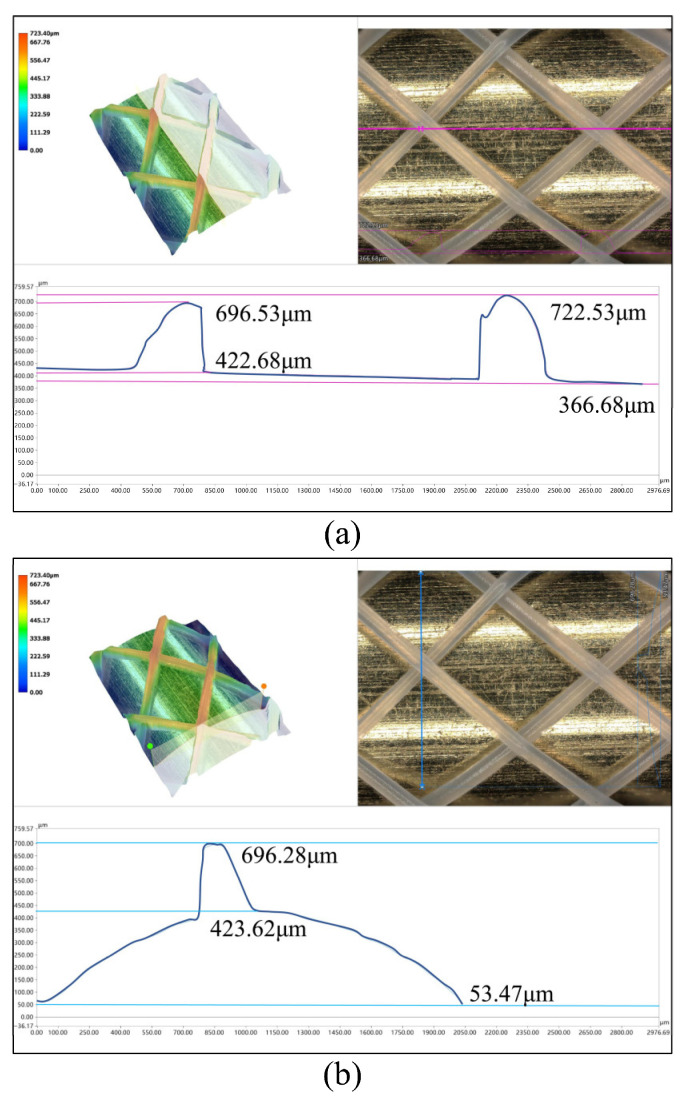
Analysis of outer contour characteristic dimensions of vascular scaffolds: (**a**) along the axial direction; (**b**) along the radial direction.

To quantify the outer contour characteristics of 3D-printed vascular scaffolds, the specimen fabricated on a 5 mm mandrel (Figure 2b) was selected for analysis. Localized observation and measurement were performed using a Keyence VHX-7100 deep-field microscope (KEYENCE CORPORATION, Osaka, Japan). Figure 3 presents multidimensional size analysis of the fabricated fully degradable polymer scaffold microstructure, organized into axial and radial measurement sets, each comprising (i) 3D topography, (ii) optical micrograph, and (iii) height profile curve. In Figure 3a,b (top–left), 3D topography maps encoded with chromatic height scales reveal stereometric features, where color gradients indicate surface undulations, preliminarily confirming uniform grid patterning, consistent fiber diameters, and stable junction morphologies. Top-right optical micrographs display planar views with measurement paths demarcated by pink/blue lines, clearly visualizing fiber crossing patterns and mesh architecture. Bottom panels in Figure 3a show height profiles along axial paths (pink lines), with maxima at fiber junctions and minima at the mandrel surface.

At the first fiber intersection point, the maximum height is 696.53 μm and the minimum is 422.68 μm, yielding a height difference of 273.85 μm. At the second fiber intersection point, the maximum height is 722.53 μm and the minimum is 366.68 μm, with a height difference of 355.85 μm. A notable discrepancy exists between the height differences at the two fiber intersection points. Analysis suggests this is due to errors in the selection of the highest positions of the fiber intersection points marked by the pink calibration lines.

The height profile curve measured along the radial blue line is presented below Figure 3b. The maximal contour height, analogous to the axial measurement, localizes at the fiber intersection points, reaching 696.28 μm, whereas the measured value at the outer contour surface of the deposition mandrel at this intersection is 423.62 μm. This yields a height differential of 272.66 μm at the fiber intersection, which corresponds to the identical anatomical position marked as the first fiber intersection in Figure 3a. The close agreement between the height differentials measured along the axial and radial axes at the same intersection demonstrates the dimensional consistency and process stability of the printing technique.

### 3.2. Microstructural Characterization

**Figure 4:**
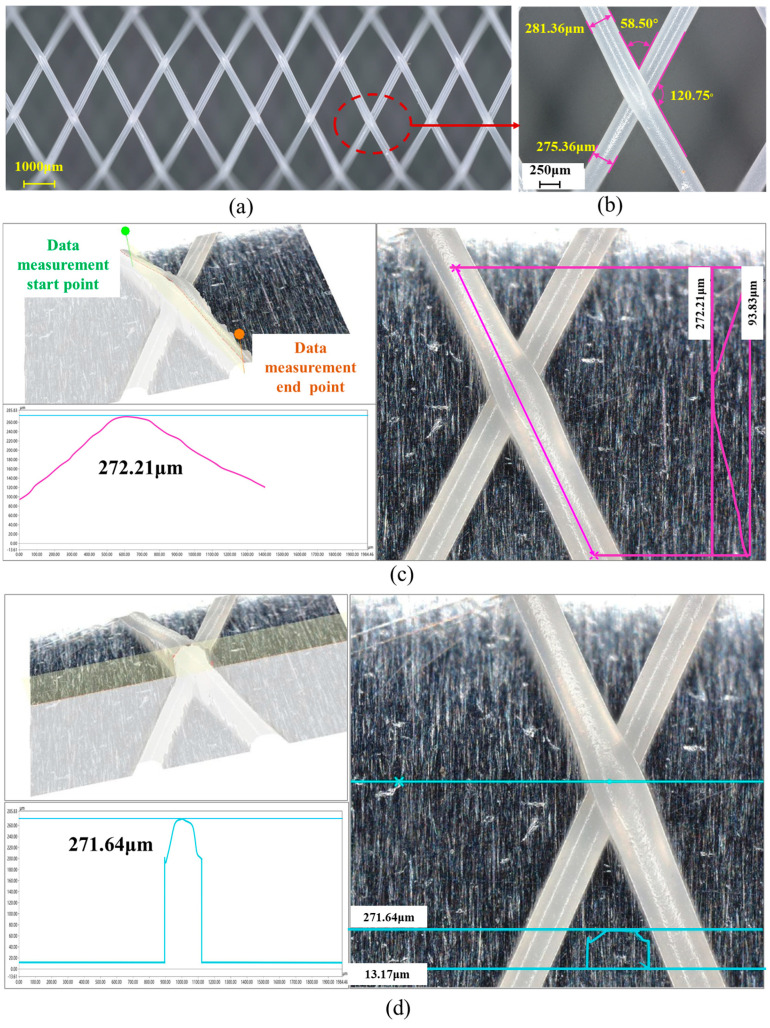
Microstructural characterization and analysis at vascular scaffold fiber junctions: (**a**) Planar view of scaffold microstructure; (**b**) Magnified local view; (**c**) Height profile curve at fiber junction; (**d**) Cross-sectional contour curve at fiber junction.

To further analyze the microstructural features of the 3D-printed vascular scaffolds, the scaffold fabricated using an 8 mm-diameter deposition mandrel (Figure 2a) was selected as the analysis specimen. The microstructure was observed and measured at 100× magnification using a Keyence VHX7100 ultra-depth-of-field digital microscope (KEYENCE CORPORATION, Osaka, Japan), as shown in Figure 4a. This planar view of the scaffold’s microstructure reveals a typical grid morphology composed of intersecting fibers forming regular diamond-shaped patterns, demonstrating the positional accuracy and process stability of the printing technique. A specific region outlined by the red dashed circle—where fiber intersection details critically influence the scaffold’s mechanical properties—was selected for further analysis.

Figure 4b presents a 500× magnification view of the red-circled region in Figure 4a. Precise measurements show that the widths of the two intersecting fibers are 281.36 μm and 275.36 μm, with crossing angles of 58.50° and 120.75°. The precision of fiber width and angle significantly affects the scaffold’s radial supporting force and flexibility. Optimal crossing angles ensure the scaffold can adapt to physiological vascular deformation while maintaining patency, thereby avoiding stress concentration.

Figure 4c displays a height profile curve of the fiber intersection node, measured along the pink-marked path following the fiber deposition direction. This curve exhibits a height peak of 272.21 μm, reflecting the thickness at the fiber intersection. Figure 4d shows the profile curve of the cross-sectional plane at the fiber intersection node, with a height peak of 271.64 μm. The close agreement between the measurements in Figure 4c,d demonstrates the uniformity and consistency of the vascular scaffold’s microarchitecture.

### 3.3. Testing Principle of Mechanical Properties

The mechanical properties of fully degradable vascular stents represent core indicators for their clinical safety and efficacy, requiring a balance of short-term support (prevention of vascular collapse), long-term matching (synchronous degradation with tissue repair), fatigue resistance (tolerance to pulsatile loading), and surgical operability. These mechanical characteristics are subject to the coupled influence of multiple factors, including stent material, structural geometry, fabrication processes, and in vivo environmental conditions. Following the additive manufacturing of vascular stents, systematic testing and characterization of their mechanical performance serve as a critical basis for evaluating and optimizing the printing process.

**Figure 5:**
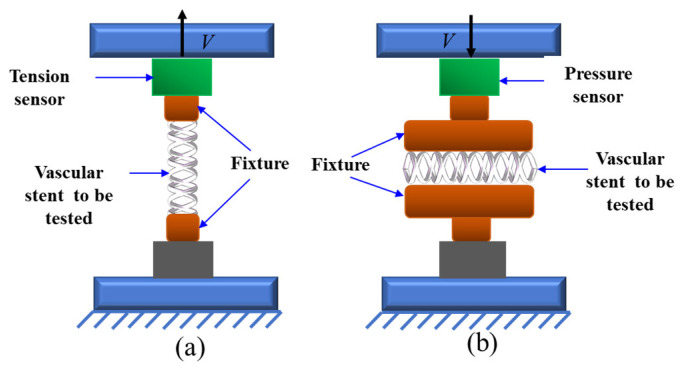
Schematic diagrams of mechanical property testing devices for vascular scaffolds: (**a**) schematic of tensile test; (**b**) schematic of flat plate compression test.

Figure 5 illustrates schematic diagrams of two experimental setups for testing the mechanical properties of vascular scaffolds. Figure 5a depicts the tensile test apparatus, which consists of the following components arranged from bottom to top: a base fixing structure, a lower fixture for securing the vascular scaffold, the scaffold under test, an upper fixture, a tension sensor, and a top-mounted moving component (the arrow labeled V− indicates its direction of motion, applying tensile force to the scaffold when moving upward). This setup measures the force exerted on the scaffold during stretching via the tension sensor to evaluate its tensile resistance. Figure 5b shows the schematic of the flat plate compression test device, with components arranged sequentially from bottom to top as: base fixing structure, lower fixture, scaffold specimen, upper fixture, pressure sensor, and a top moving component (the arrow labeled V- denotes its motion direction, applying compressive force to the scaffold when moving downward). This apparatus uses a pressure sensor to measure the force endured by the scaffold during compression, thereby assessing its compressive performance.

For the mechanical tests, each diameter group of vascular stents (3 mm, 4 mm, 5 mm, 8 mm) included three replicate specimens. All specimens were fabricated via identical manufacturing processes and subjected to uniform experimental conditions to ensure data comparability. Following tensile and compression tests, the standard deviation (SD) and standard error (SE) were calculated for all mechanical parameters to characterize data variability and precision.

### 3.4. Tensile Test and Analysis

**Figure 6:**
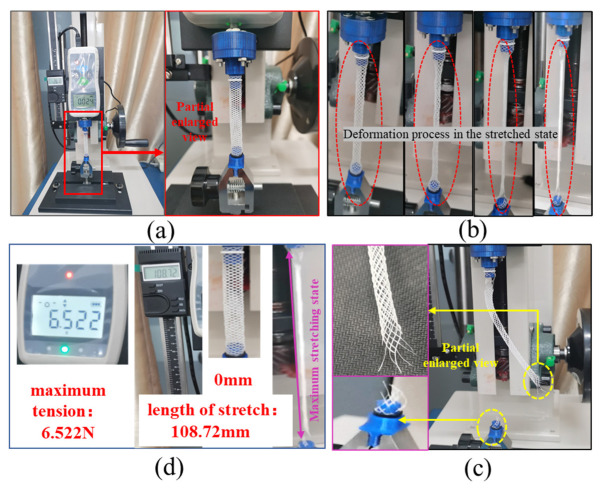
Tensile testing procedure for vascular scaffolds: (**a**) Experimental setup preparation; (**b**) Tensile loading process; (**c**) Post-failure morphology and fracture surface; (**d**) Experimental data output.

Figure 6 illustrates the experimental process for tensile testing of vascular scaffolds. An EDIBURG force tester was employed as the testing machine, with an 8 mm-diameter vascular scaffold as the specimen. The tester is equipped with a high-precision force sensor and displacement measurement device to record real-time mechanical data and displacement changes during stretching. The testing procedure was as follows: First, the vascular scaffold was mounted on the tensile tester, ensuring both ends were securely clamped in the machine’s fixtures (Figure 6a). A constant tensile rate was then applied to exert pulling force on the scaffold.

During the stretching process, a high-speed camera captured multi-angle images of the scaffold to record morphological changes at different deformation stages. The red dashed frame in Figure 6b highlights the structural deformation under tension, showing that as the tensile force increased, the scaffold’s diameter gradually decreased while its length increased. Fracture initiated at the junction with the bottom fixture. Figure 6c depicts the post-fracture state and fracture surface details, revealing significant deformation of the scaffold: the originally regular mesh structure was elongated and twisted, losing its initial geometric integrity. Close examination of the magnified image shows broken filaments at the yellow-dashed frame, with some filaments detached from their connection points, causing damage to the mesh structure.

Throughout the test, the force tester continuously recorded tensile force and displacement data. As shown in Figure 6d, the maximum tensile force measured was 6.522 N, and the maximum elongation of the scaffold reached 108.72 mm.

**Figure 7:**
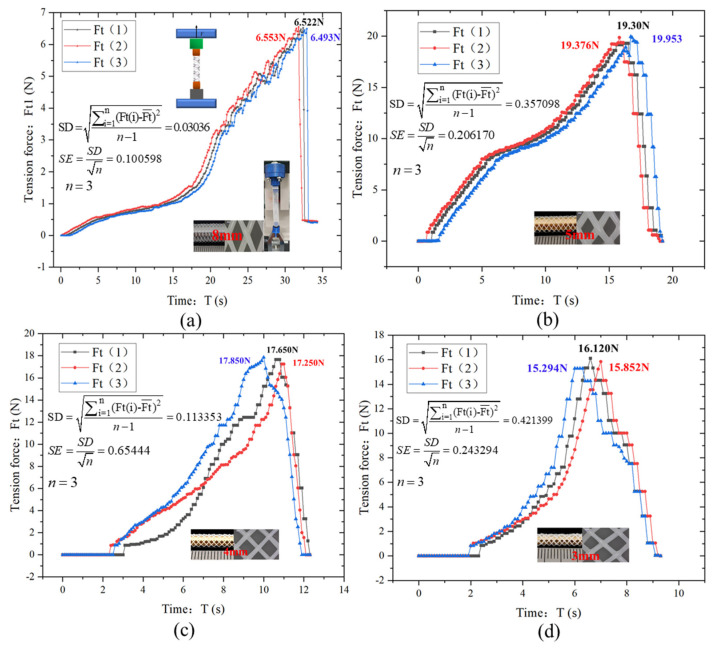
Tensile Force Variation Curve of Vascular Scaffold during Tensile Test: (**a**) Measurement data of three samples with a diameter of 8 mm; (**b**) Measurement data of three samples with a diameter of 5 mm; (**c**) Measurement data of three samples with a diameter of 4 mm; (**d**) Measurement data of three samples with a diameter of 3 mm.

Figure 7 shows the tensile force (Ft)-time (T) curves of tensile tests on vascular stent samples with four different diameters (8 mm, 5 mm, 4 mm, 3 mm). Each group of tests was repeated 3 times to verify the reliability of the results, and the data dispersion was quantified by standard deviation (SD) and standard error (SE).

The curve exhibits an overall upward trend, indicating that the tensile force increases continuously until reaching a peak, after which a distinct change occurs. A stage-wise analysis is as follows: Initial Stage: at the onset, the tensile force remains low and grows gradually. This phenomenon arises because the scaffold’s microstructure initially undergoes adaptive adjustment to external loading, with all structural components not yet fully engaged in the stretching process. Internal stress within the material is still in the accumulation phase, and the scaffold demonstrates minimal resistance to deformation as molecular chains and fiber intersections gradually align with the tensile direction. Rapid Growth Stage: during this phase, the tensile force rises sharply with a steeply increasing curve slope. This reflects the rapid accumulation of internal stress as the scaffold material enters the elastic deformation regime. Molecular chains and microfibers, previously in a coiled or random configuration, are progressively stretched and aligned along the tensile axis, significantly enhancing the material’s load-bearing capacity. The linear relationship between force and displacement during this stage confirms typical elastic behavior, where deformation is reversible and directly proportional to the applied load. Near-Peak Stage: the upward trend of the curve diminishes, approaching a plateau as the force nears its maximum value. This signifies that the material is approaching its elastic limit or yield point, where microstructural deformation (e.g., fiber stretching, interface slippage) reaches a critical threshold. The decreasing rate of force increase indicates that the material’s ability to resist further deformation is waning, with energy dissipation mechanisms (e.g., plastic deformation at fiber junctions) beginning to dominate. Peak and Post-Failure Stage: the tensile force peaks, after which the curve experiences a precipitous decline. This abrupt drop corresponds to the scaffold reaching its ultimate tensile strength, leading to catastrophic failure—manifested as filament breakage, fiber-matrix debonding, or structural collapse. The sudden loss of load-bearing capacity highlights the brittle failure characteristics of the scaffold material under uniaxial tension, with failure initiating at stress-concentrated regions.

For the 8 mm diameter stent (Figure 7a), the three repeated curves show an extremely high degree of fitting. The tensile force first rises slowly over 15 s, then enters a rapid growth phase, and finally reaches a stable value of approximately 6.5 N at 30 s. Its SD = 0.0306 and SE = 0.0107 are the smallest among the four groups, reflecting that the large-diameter stent has strong structural rigidity, uniform deformation, and excellent test repeatability. For the 5 mm diameter stent (Figure 7b), the tensile force growth rate increases significantly after 10 s, and the final stable tensile force reaches 19.3 N (the highest among the four groups). Although SD = 0.3580 and SE = 0.2067 are slightly higher than those of the 8 mm group, they are still within a controllable range, indicating that the medium-diameter stent achieves a good balance between structural density and load-bearing capacity. For the 4 mm diameter stent (Figure 7c), the rapid growth phase advances to 5 s, the tensile force peak drops to 17.85 N, and the curve in the stable phase shows slight fluctuations, corresponding to moderate dispersion with SD = 0.1135 and SE = 0.0654. This is related to the uneven local deformation caused by the decreased stiffness of the stent at this diameter. For the 3 mm diameter stent (Figure 7d), the rapid growth phase further advances to 3 s, the stable tensile force drops to 16.12 N, the curve fluctuations are the most significant, and SD = 0.4214 is the largest among the four groups. This is due to the excessively high structural flexibility of the small-diameter stent, which amplifies individual differences in the tensile deformation mode. Overall, the stable tensile force of the stent shows a “first rising then falling” trend as the diameter decreases (with 5 mm being optimal), while the dispersion of mechanical properties gradually increases as the diameter decreases. This regularity provides an experimental basis for the size design and process optimization of stents adapted to blood vessels of different diameters.

**Figure 8:**
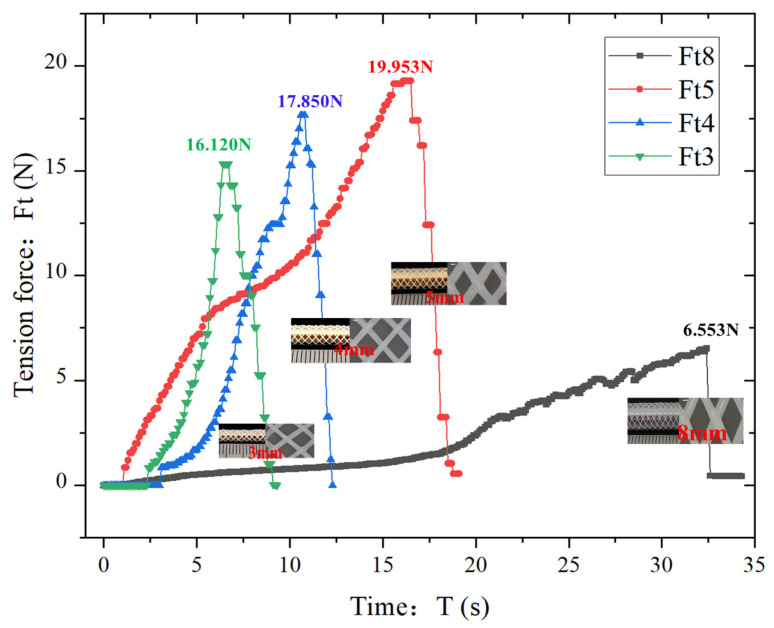
Comparison chart of tensile force change curves of vascular stents with different diameters in tensile tests.

Figure 8 presents the tensile force (*F_t_*) vs. time (*T*) curves for these specimens with different diameters. Peak Force and Time-to-Peak Trends: The 3 mm scaffold (*F_t_*_3_) reached a peak tensile force of 16.12 N at 7 s, followed by abrupt force decline. The 4 mm scaffold (*F_t_*_4_) exhibited a higher peak force of 17.85 N at 11 s. The 5 mm scaffold (*F_t_*_5_) demonstrated the highest peak force of 19.53 N at 17 s. In contrast, the 8 mm scaffold (Figure 7) reached its peak force of 6.523 N at 32 s, the longest time-to-peak among all specimens. Diameter-Dependent Mechanical Behavior: As scaffold diameter increased from 3 mm to 5 mm, peak tensile force increased by 26.5%, while time-to-peak extended from 7 s to 17 s. However, the 8 mm scaffold showed a paradoxical decrease in peak force (−66.2%) despite the longest time-to-peak. Mechanism Analysis: 8 mm Scaffold: Its larger grid geometry and sparser fiber distribution (Figure 2a) allowed greater tensile deformation, hence the prolonged time-to-peak. However, the expanded mesh structure reduced interfacial bonding density, leading to premature structural failure at lower forces due to stress concentration at sparse fiber intersections. 5 mm Scaffold: The denser grid architecture (Figure 2c) and enhanced interfacial bonding strength enabled more effective stress dissipation across the scaffold matrix. The optimal balance of fiber packing density and geometric uniformity facilitated progressive load transfer, delaying failure and increasing peak force. Diameter-Microstructure-Mechanics Correlation: Reduced diameter correlates with increased fiber weaving density (Figure 2d), which shortens stress transmission paths and enhances load-bearing capacity. Conversely, excessive diameter (e.g., 8 mm) compromises structural integrity due to insufficient inter-fiber bonding points, despite higher deformation capacity.

Microstructural parameters including fiber packing density, grid geometry, and interfacial bonding strength are key determinants of tensile performance. While larger-diameter scaffolds exhibit higher deformation tolerance, their mechanical strength is compromised by sparse architectures. Smaller-diameter scaffolds (3–5 mm) demonstrate superior tensile resistance due to denser microstructures, highlighting the critical role of design parameters in optimizing stent mechanics for target vessel applications.

### 3.5. Analysis of Tensile Fracture Morphology and Mechanism

**Figure 9:**
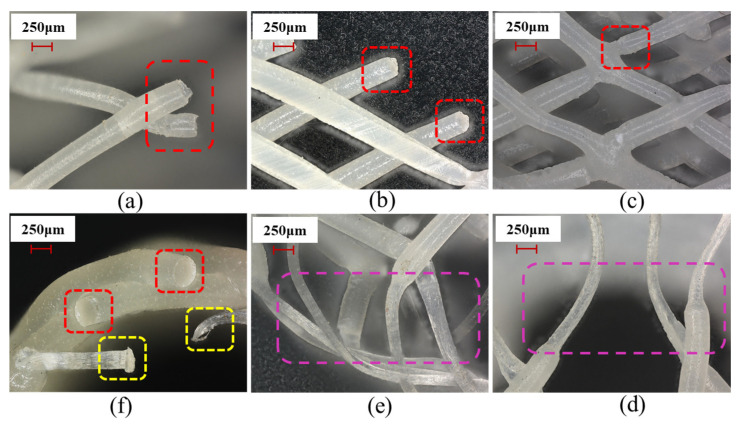
Microscopic Morphology of Vascular Scaffold after Tensile Fracture: (**a**) Brittle Fracture Zone 1; (**b**) Brittle Fracture Zone 2; (**c**) Node Fracture Zone; (**d**) Plastic Deformation Zone 1; (**e**) Plastic Deformation Zone 2; (**f**) Complex Fracture Zone.

In summary, the tensile fracture morphology of PCL vascular scaffolds is jointly regulated by the material’s intrinsic properties and the scaffold’s geometric structure (e.g., stress concentration, multi-directional loading). Brittle fracture (Figure 9a–c) mostly occurs in stress-concentrated areas (nodes and intersections within the red box), where stress concentration causes brittle failure without necking (flat ends), indicating that the structure restricts molecular chain slip and inhibits necking. Ductile fracture (Figure 9d,e), more prominent in long straight filaments or interwoven structures, reflects PCL’s molecular chain slip ability, with uniform diameter reduction during stretching and surface texture changes (traces of molecular chain slip within the purple box), demonstrating high-toughness deformation capacity. Curved structures (Figure 9f) induce ductile–brittle transitions(within the yellow box), reflecting material responses under complex stresses. These results provide micro-mechanical insights for optimizing scaffold design (e.g., reducing stress-concentrated nodes and regulating material toughness).

### 3.6. Compression Test and Analysis

**Figure 10:**
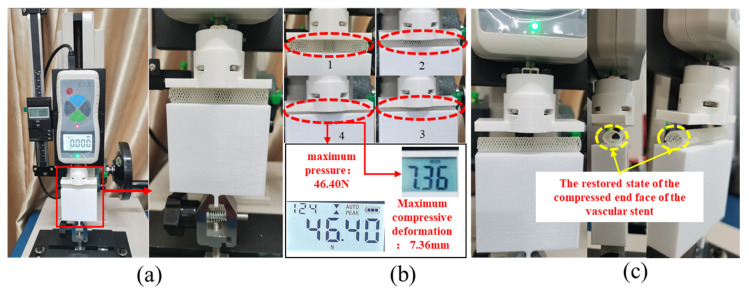
Experimental process of flat plate compression test for vascular scaffolds: (**a**) experimental preparation and specimen installation; (**b**) compression process and data measurement; (**c**) post-compression recovery state.

Figure 10 illustrates the flat plate compression test procedure for vascular scaffolds, using an EDIBURG force tester as the testing machine and an 8 mm-diameter scaffold as the specimen. The tester is equipped with a high-precision force sensor and displacement measurement device to record real-time mechanical data and displacement changes during compression. The testing process is as follows: The vascular scaffold was placed between the fixtures of the force tester, ensuring it was centrally positioned and stable. At the initial state, the force sensor reading was 0.000 N, and the displacement data was 0 mm. The upper platen of the tester was driven downward at a constant rate to compress the scaffold. Figure 10b shows the scaffold’s morphological changes at different compression stages. The red dashed frame highlights the gradual flattening of the scaffold. The force sensor continuously collected pressure data until reaching a maximum pressure of 46.40 N, at which point the maximum compression deformation of the scaffold was 7.36 mm. After reaching the predetermined compression stage, loading was halted, and the upper platen was retracted. Observation of the area marked by the yellow dashed frame in Figure 10c revealed that the scaffold exhibited partial rebound recovery, but residual deformation remained compared to its initial state. This indicates that the compression process involved both elastic deformation and partial plastic deformation in the scaffold.

**Figure 11:**
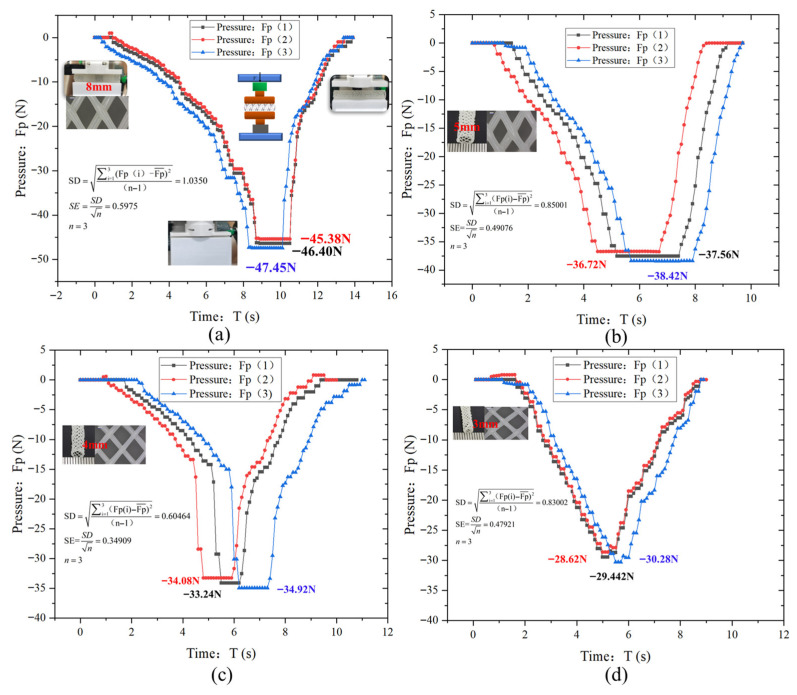
Pressure Variation Curve of Vascular Scaffold during Compression Test. (**a**) Measurement data of three samples with a diameter of 8 mm; (**b**) Measurement data of three samples with a diameter of 5 mm; (**c**) Measurement data of three samples with a diameter of 4 mm; (**d**) Measurement data of three samples with a diameter of 3 mm.

Figure 11 depicts the variation in compression pressure (*F_p_*) with time (*T*) for the vascular scaffold with four different diameters (8 mm, 5 mm, 4 mm, 3 mm). Each group of tests was repeated 3 times to verify the reliability of the results, and the data dispersion was quantified by standard deviation (SD) and standard error (SE).

An overall analysis shows that all curves first drop to the lowest point and then rise, which reflects the dynamic pressure changes during the compression and unloading processes. A stage-wise analysis is as follows: the curve starts with a pressure value close to 0 N, and pressure decreases rapidly over time. This occurs because the testing machine’s platens begin applying compressive force to the scaffold at the initial compression stage. As the scaffold structure is gradually compressed, its internal microstructure deforms to resist the external load, causing the recorded pressure value to decrease (negative sign denotes compressive direction). This indicates that the compressive load borne by the scaffold increases continuously. The curve reaches its lowest point, signifying the scaffold is compressed to its maximum extent. At this stage, the internal structure bears the ultimate compressive load, and material deformation reaches the maximum under current test conditions, reflecting the scaffold’s capacity to withstand peak compressive loading. During this period, the positions of the upper and lower platens are fixed to maintain constant compression, resulting in a stable pressure value with no further change. The curve begins to rise as the pressure value gradually rebounds toward 0 N. This phenomenon occurs when the testing machine stops compressing and initiates unloading, allowing the elastic potential energy stored within the scaffold to be released. The structural rebound leads to a gradual reduction in compressive pressure, demonstrating the scaffold’s elastic recovery characteristics.

For the 8 mm diameter samples (Figure 11a), the three curves show an extremely high degree of fitting. They remain stable in the initial stage, then drop rapidly, and finally stabilize at a pressure of approximately −47.45 N, with a corresponding SD = 1.0530 and SE = 0.5995. The low dispersion indicates that the large-diameter stent has high structural rigidity, uniform deformation, and excellent test repeatability. For the 5 mm samples (Figure 11b), the curves show a consistent trend, with a stable pressure of approximately −38.42 N, SD = 0.85001, and SE = 0.49076. The dispersion slightly increases but remains at a low level, indicating good load-bearing capacity and consistency. For the 4 mm samples (Figure 11c), the dispersion of the curves increases, with a stable pressure of approximately −34.92 N, SD = 0.60646, and SE = 0.34909. The decreased structural rigidity leads to uneven deformation and increased dispersion. For the 3 mm samples (Figure 11d), the curves show the most significant differences, with a stable pressure of approximately −30.28 N, SD = 0.83300, and SE = 0.47921. The small-diameter stents have high flexibility, which amplifies individual differences in deformation modes and results in the highest dispersion. Overall, the compressive load-bearing capacity of the stents increases with the increase in diameter, and the data dispersion increases with the decrease in diameter. This regularity provides an experimental basis for the size design and process optimization of stents with different tube diameters.

**Figure 12:**
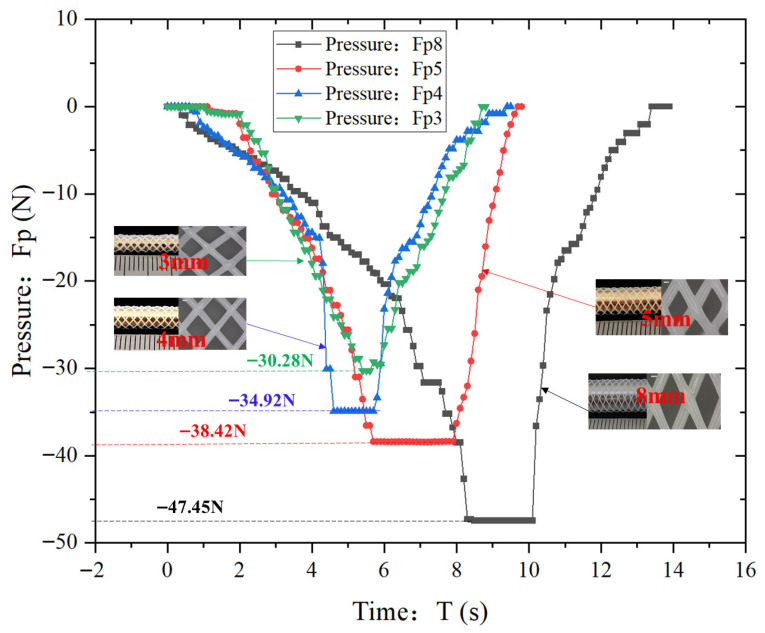
Comparison chart of Pressure Variation Curve of Vascular Scaffold with different diameters in Compression Test.

Figure 12 depicts the variation in compression pressure (*Fp*) with time (*T*) for the vascular scaffold with four different diameters (8 mm, 5 mm, 4 mm, 3 mm). Each group of tests was repeated 3 times to verify the reliability of the results, and the data dispersion was quantified by standard deviation (SD) and standard error (SE).

All four curves exhibit a trend of first decreasing and then increasing, which reflects the dynamic pressure variations during the compression and unloading processes. In the compression phase, the pressure values are negative with their absolute values gradually increasing, indicating that the compressive load borne by the stents continuously rises. In the unloading phase, the pressure values gradually rebound to near 0 N, demonstrating the elastic recovery property of the stents.

At the start of compression, the pressure values of all four curves are close to 0 N. As time progresses, the platen of the testing machine applies pressure to stents of different specifications, and the stent structures are gradually compressed; the decreasing trends of the four curves differ: the Fp8 curve decreases most steeply, followed by the relatively steep decline of the Fp5 curve, while the Fp3 and Fp4 curves decrease more gently. The compressive ultimate load (minimum pressure) of each stent shows a distinct diameter dependence: the minimum pressures of the 8 mm, 5 mm, 4 mm, and 3 mm stents are −47.45 N, −38.42 N, −34.92 N, and −30.28 N, respectively. This indicates that the compressive load-bearing capacity of the stents increases significantly with increasing diameter, which is directly associated with the higher structural stiffness of larger-diameter stents.

During the unloading process, all four curves start to rise, and the pressure values gradually rebound. This occurs because after the testing machine ceases compression and initiates unloading, the elastic potential energy stored in the stents is released, prompting structural rebound. The rebound trends of the curves also vary: the 8 mm stent exhibits the most significant rebound trend, followed by the 5 mm stent, while the 3 mm stent has the smallest rebound amplitude. This discrepancy stems from the varying degrees of residual deformation in stents of different diameters: smaller-diameter stents possess higher structural flexibility, making them prone to more inelastic residual deformation during compression. This reduces the storage and subsequent release of elastic potential energy, ultimately resulting in diminished rebound capability.

Correlating with the repeated test curves in Figure 11, it can be further confirmed that the compressive load-bearing capacity, elastic recovery property, and consistency of mechanical performance of vascular stents are all positively correlated with diameter. This conclusion provides direct mechanical experimental evidence for the size design, structural optimization (e.g., stiffness enhancement design for small-diameter stents), and clinical selection of stents adapted to blood vessels of different diameters.

### 3.7. Quantitative Comparison of Mechanical Property Indicators

This paper selects 2 core mechanical performance indicators (tensile strength and radial support strength) for the clinical application of PCL vascular stent BVS. Based on international standards, industry norms, and the physiological mechanical characteristics of clinical radial arteries, a systematic comparison table is constructed among commercial BVS standards, clinical radial artery mechanical requirements, and the research results of this paper. The advantages and adaptability of this research are clarified through quantitative differences. The calculation method adopted refers to Formulas (1) and (2). A 5 mm diameter PCL vascular stent formed by printing is selected as the calculation sample, and the maximum tensile breaking force and maximum bearing compression force are taken as the calculation data. The specific comparisons are as follows in Table 2:

## 4. Discussion

This study systematically tested the core mechanical properties of polycaprolactone (PCL) vascular stents, clarified their clinical mechanical compatibility, sorted out research limitations, and proposed optimization directions, providing key references for clinical translation. Radial support strength is a core indicator for preventing and controlling vascular retraction and restenosis. The standard for commercial bioresorbable vascular stents (BVS) is ≥150 kPa, and the clinical threshold for radial artery systolic pressure is 180 kPa as the minimum requirement. The radial support strength of the PCL stent in this study reached 244.6 ± 5.3 kPa, which is 63.1% higher than the commercial standard and higher than the clinical threshold. It can effectively resist the periodic pressure of blood vessels and reduce the risk of collapse and restenosis. At the same time, the flexibility of PCL material endows the stent with elastic buffering capacity, which can reduce mechanical stimulation to the radial artery wall, reduce inflammatory reactions, and improve clinical safety. Tensile tests showed that the stable tensile force of stents with 4 diameters (8 mm, 5 mm, 4 mm, 3 mm) first increased and then decreased as the diameter decreased (5 mm > 4 mm > 3 mm > 8 mm), which was due to the coupling effect of “diameter-structural stiffness-bearing capacity”. Moreover, the smaller the diameter, the greater the data dispersion, suggesting that the performance consistency of small-diameter stents is easily affected by the process or deformation mode. The tensile strength of the stent is 45.1 ± 0.81 MPa, which is 50.3% higher than the commercial standard (≥30 MPa), meeting the mechanical requirements of clinical operations, avoiding the risk of fracture during implantation, and verifying the rationality of the design.

In conclusion, the core mechanical indicators of the PCL stent in this study exceed the commercial BVS standards, are compatible with the physiological characteristics of clinical radial arteries, and have especially optimized fatigue performance and retraction rate, overcoming the mechanical shortcomings of traditional bioresorbable stents. Through the collaborative optimization of “material-structure-performance”, the precise matching of mechanical properties and clinical needs is achieved, providing solid support for clinical translation.

This study has limitations: first, static loading did not simulate the human physiological environment and dynamic loads, so the conclusions cannot fully reflect the real in vivo response; second, the evaluation dimension is limited to mechanical properties and does not cover core safety indicators such as biocompatibility; third, the sample size is small and does not include multiple production batches, so the universality is limited; fourth, there is a lack of clinical anatomical compatibility analysis, resulting in insufficient clinical pertinence. Subsequent supplementary studies will be carried out: building a dynamic platform to simulate in vivo conditions; improving the multi-dimensional evaluation system and supplementing biocompatibility tests; expanding the sample size to cover multiple batches; and conducting compatibility tests combined with clinical vascular anatomy data. Through the above studies, the shortcomings will be addressed, the evaluation system will be improved, and more comprehensive and reliable basis will be provided for the clinical translation of the stent.

## 5. Conclusions

This study developed an electric-field-driven melt-spinning rotational deposition 3D printing technology. By precisely regulating the synergistic interplay between electric field force and surface tension, it achieved high-precision fabrication (fiber diameter deviation < 5%) of fully biodegradable polycaprolactone (PCL) vascular scaffolds spanning diameters of 3–8 mm, overcoming key technical bottlenecks in personalized forming and microstructural control of degradable stents. Reducing the scaffold diameter from 8 mm to 3 mm significantly increased fiber weaving density and mesh count per unit area, enhancing cell attachment and tissue integration potential. Concurrent synergistic optimization of scaffold strength enabled the fulfillment of mechanical adaptation requirements for diverse vasculature. Tensile testing revealed that the 5 mm-diameter scaffold exhibited a 38% increase in peak tensile force compared to the 8 mm counterpart, attributable to its higher interfacial bonding strength and denser mesh architecture. Micro-fracture analysis elucidated the underlying synergistic mechanism: stress concentration at nodal points induced brittle fracture, while molecular chain slippage in longer fiber segments governed ductile deformation. Conversely, the 8 mm-diameter scaffold demonstrated superior compressive performance, with a 20% increase in load-bearing capacity and a 92% elastic recovery rate, significantly outperforming smaller-diameter structures. These unique mechanical properties provide key evidence for the design of customized personalized stents, thereby helping to optimize the long-term safety of implantable devices.

## Figures and Tables

**Table 1:** Process parameters of forming experiment.

Parameter	Value	Parameter	Value
Printing material	PCL	Air pressure (*P_r_*)	15 kPa
Temperature of crucible (*T_C_*)	150 °C	Diameter of deposition mandrel (*D_m_*)	8 mm, 5 mm, 4 mm, 3 mm
Diameter of nozzle (*D_n_*)	300 μm	Motion speed of nozzle (*V*)	5 mm/s
Deposition height (*H*)	2 mm	Rotation speed of axis (*ω*)	16 rad/s
Electric field voltage (*U*)	1.8K v	Temperature of nozzle (*T_n_*)	90 °C

**Table 2:** Quantitative comparison table of performance indicators.

Performance Indicators	Commercial BVS Standards	Clinical Radial Artery Mechanical Requirements (Adult Normal Physiological State)	The Research Results of this Paper (PCL Vascular Stent)	Quantification of Differences from Commercial Standards	Compatibility with Clinical Requirements
Radial support strength (Rs: kPa)	≥150	≥180 (Withstand the peak systolic blood pressure, corresponding to 140 mmHg)	244.6 ± 5.3	63.1% above the standard	Fully compatible with sufficient redundancy support
Tensile Strength ($\sigma_{t}$: MPa)	≥30	≥35 (Higher than the radial artery wall strength of 25–35 MPa, ensuring operational safety)	45.1 ± 0.81	50.3% above the standard	Fully compatible with excellent resistance to operational damage

## Data Availability

The original contributions presented in this study are included in the article. Further inquiries can be directed to the corresponding authors. All the supplementary data to this article reported here can be made available on request by email (chaoyanpu@163.com).

## Abbreviations

The following abbreviations are used in this manuscript:

PCL	Polycaprolactone
CAD	Coronary Artery Disease
PCI	Percutaneous Coronary Intervention
BPVS	Biodegradable Polymeric Vascular Scaffolds
BMS	Bare Metal Stents
*DES*	Drug Eluting Stents
*SLM*	Selective Laser Melting
*SLA*	Stereolithography
*FDM*	Fused Deposition Modeling
*PLA*	Polylactic Acid
*PGA*	Polyglycolic Acid
*F_N_*	Normal electric field force
*F_T_*	Tangential electric field force
*F_E_*	Electric polarization force
*F_s_*	Resistive forces of surface tension
*Fv*	Viscous drag
*F_D_*	Viscous drag force
*CCD*	High speed charge coupled device
*DC*	High voltage direct current
*PID*	Proportional Integral Derivative
*P_r_*	Air pressure
*T_C_*	Temperature of crucible
*D_m_*	Diameter of deposition mandrel
*D_n_*	Diameter of nozzle
*V*	Motion speed of nozzle
*H*	Deposition height
*ω*	Rotation speed of axis
*U*	Electric field voltage
*Tn*	Temperature of nozzle
*F_P_*	Compression pressure
*T*	Time
$\sigma_{t}$	Tensile Strength
*Rs*	Radial Support Strength

$\sigma_{t}$
